# The Importance of Coaches’ Autonomy Support in the Leisure Experience and Well-Being of Young Footballers

**DOI:** 10.3389/fpsyg.2018.00840

**Published:** 2018-05-29

**Authors:** Isabel Balaguer, Isabel Castillo, Ricardo Cuevas, Francisco Atienza

**Affiliations:** ^1^Faculty of Psychology, Universitat de València, Valencia, Spain; ^2^Faculty of Education, Universidad de Castilla-La Mancha, Ciudad Real, Spain

**Keywords:** leisure experience, well-being, coach-autonomy support, needs satisfaction, intrinsic motivation, soccer

## Abstract

Drawing on the self-determination framework, the study examined the effect of coaches’ autonomy support on the leisure experience of young male football players. Specifically, a model was tested analyzing the long-term predictive power of the players’ perceptions of the coaches’ autonomy support at the beginning of the season on the subjective vitality of young football players at the end of the season, through needs satisfaction and intrinsic motivation (IM). Moreover, we tested whether the effects of coaches’ autonomy support on the aforementioned variables (needs satisfaction, IM, and subjective vitality) at the end of the season remained at the beginning of the following season. Because the coach in the second season was not the same one as in the first season, the perception of coaches’ autonomy support at the beginning of the second season was used as a control variable. Three hundred and sixty football players (*M* age = 12.60 years; *SD* = 0.52) completed a questionnaire on the variables of interest at the beginning of the first season (T1), at the end of the first season (T2), and at the beginning of the second season (T3). The results of the path analyses showed that players’ perceptions of coaches’ autonomy support at the beginning of the season (T1) positively predicted needs satisfaction at the end of the first season (T2), which in turn predicted IM at the end of the first season (T2). Additionally, IM significantly and positively predicted subjective vitality at the end of the first season (T2). Finally, needs satisfaction, IM, and subjective vitality at the end of the second season (T2) positively predicted these same variables at the beginning of the second season (T3). Results emphasized the importance of the autonomy support offered by the coach in promoting the quality of young people’s leisure experience playing football and its benefits for their well-being.

## Introduction

Contemporary theories of motivation argue that the coach’s interpersonal style has a significant impact on athletes’ quality leisure experiences in sport contexts and their well-being, and that some motivational mechanisms can explain these relationships (Ryan and Deci, 2000; Duda, 2013). Based on these postulates, several studies have analyzed the motivational processes in childhood and adolescence in sports contexts, noting the importance of the climate coaches create in the motivation and well-being of young people who practice sports (Mageau and Vallerand, 2003; Duda and Balaguer, 2007; Duda et al., 2018). Grounded in the self-determination framework (SDT; Ryan and Deci, 2017), the focus of this paper is to test whether playing football in an autonomy-supportive climate maximizes the quality of the leisure experiences of young footballers and their well-being in the long term.

Young athletes spend part of their time in sport contexts, which can have benefits for their physical, psychological, and social development. One of these potential benefits is their well-being. From the eudaimonic perspective, well-being has been considered a complex construct consisting of optimal experience and functioning (Ryan and Deci, 2001). From this perspective, well-being is focused on meaning and self-realization, and defined in terms of the degree to which the person is fully functioning and witnesses personal growth. One key indicator of this construct is subjective vitality, defined as “one’s conscious experience of possessing energy and aliveness” (Ryan and Frederick, 1997). When young people feel subjective vitality, they feel good, active, with a go-ahead attitude, and interested in the development of their competencies and possibilities.

According to self-determination theory, variability in well-being can be better understood by considering the degree to which the environment satisfies people’s basic psychological needs and intrinsic motivation (IM) (Deci and Ryan, 2000; Ryan and Deci, 2000). IM refers to engaging in an activity for the inherent pleasure and satisfaction derived from the activity itself (Deci and Ryan, 1985), and it is associated with the most positive experiences and outcomes, such as well-being and optimal functioning (Ryan and Deci, 2000). Considering its favorable outcomes, IM is the most desirable form of motivation in an achievement domain, and so we have it included in our study as an antecedent of well-being.

Although motivation stems from several different sources, it is viewed in this paper from a needs-based perspective within the SDT framework (Ryan and Deci, 2017); thus, motivation is thought to be linked to athletes’ psychological needs satisfaction. That is, when athletes perceive themselves as acting with a sense of autonomy, competence, and relatedness during their sport participation, they experience a high level of motivation in their leisure experiences. According to SDT, young athletes’ feelings of competence, autonomy, and relatedness are ‘basic’ and necessary needs that should be nourished in youth sport. Autonomy refers to feeling a sense of personal causality and volition in one’s actions (DeCharms, 1968). Competence denotes feeling capable of influencing the surrounding environment in a meaningful way (White, 1959). Finally, relatedness is defined as the extent to which individuals feel a sense of belonging and connection with others in the social context (Ryan and Deci, 2000).

Self-determination theory posits that IM and needs satisfaction develop through the person’s interaction with the environment. Specifically, it has been proposed that a high quality leisure experience in youth sport is more likely when children participate in an autonomy-supportive atmosphere (Ryan and Deci, 2000). Coaches who are autonomy supportive interact with their athletes: “providing choice within specific rules and limits, providing rationales for tasks and limits, acknowledging the other person’s feelings and perspectives, providing opportunities for initiative taking and independent work, providing non-controlling competence feedback, avoiding controlling behaviors, and preventing ego-involvement” (Mageau and Vallerand, 2003, p. 886). Mageau and Vallerand consider that these behaviors together represent the autonomy-supportive interpersonal style.

Grounded in the principles of SDT (Deci and Ryan, 1985), Vallerand (1997, 2001) offered a hierarchical model of intrinsic and extrinsic motivation (HMIEM) that operates at three levels of generality: the global (or personality), contextual (or life domain as sport), and situational (or state) levels. Vallerand proposed the following sequence for each level: social factors → needs satisfaction → quality of motivation → consequences. The purpose of this study is to test the proposed motivational sequence at the contextual level, specifically in the sport context.

Research conducted in the sport setting has supported the assumed desirable motivational processes and outcomes associated with more autonomy-supportive coaching behaviors (Duda and Balaguer, 2007). For example, a number of cross-sectional studies in the sport context (e.g., Balaguer et al., 2008) have tested the relationships in the four-stage motivational sequence (perceived coach autonomy support → needs satisfaction → motivation → psychological well-being). In a study with competitive athletes from various sports, Balaguer et al. (2008) stated that the perception of coaches’ autonomy support corresponded to greater satisfaction of the need for autonomy and relatedness. They also observed that the more competent, autonomous, and related the athletes felt, the higher their self-determined motivation and, in turn, their well-being (self-esteem and life satisfaction). In other cross-sectional studies (Adie et al., 2008; López-Walle et al., 2012), researchers have tested models with a three-stage motivational sequence: social factors → needs satisfaction → well-being, a sequence that corresponds to the basic psychological needs theory. In both studies, Adie et al. (2008) and López-Walle et al. (2012) found that coaches’ autonomy support predicted participants’ basic needs satisfaction for autonomy, competence, and relatedness. In turn, basic needs satisfaction predicted well-being indicators.

Basic Psychological Needs Theory has also been tested in longitudinal studies (Adie et al., 2012; Balaguer et al., 2012; González et al., 2017). In three longitudinal studies, two with measures at the beginning and end of a season (Balaguer et al., 2012; González et al., 2017) and one with six measures throughout two competitive seasons (Adie et al., 2012), The authors found that the perception of coaches’ autonomy support predicted needs satisfaction, which, in turn, predicted subjective vitality in young footballers. In addition, in a study by González et al. (2016) with young male athletes and measures taken at the beginning and end of two consecutive seasons, the results revealed that changes in coaches’ autonomy support predicted changes in needs satisfaction, which, in turn, positively predicted changes in self-esteem within the same season. In sum, there is evidence from both cross-sectional and longitudinal studies indicating that autonomy-supportive coaches enhance athletes’ IM and well-being because coaches support athletes’ autonomy, competence, and relatedness needs satisfaction during their coaching.

**FIGURE 1:**
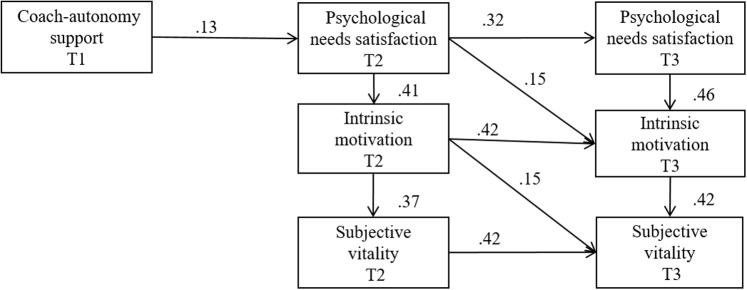
Associations between perceived coach autonomy support, psychological needs satisfaction, intrinsic motivation, and subjective vitality over time. All paths are standardized and significant (*p* < 0.01). T1, beginning of the first season; T2, end of the first season; T3, beginning of the second season. Control variables are not depicted in this figure for clarity of the presentation.

The aim of this study is to test the effect of the perception of coaches’ autonomy support at the beginning of the season (T1) on subjective vitality at the end of the first season (T2) through needs satisfaction and IM at the end of the first season (T2), and whether the effects on the variables at the end of the season (T2) remain at the beginning of the following season (T3). The predictions embedded in the model being tested are part of a recent consensus statement on the optimal causes and benefits of young people’s engagement in physical activities in their leisure time (Bangsbo et al., 2016). Specifically, and in line with previous research (e.g., Adie et al., 2012; Balaguer et al., 2012; González et al., 2016, 2017), it is hypothesized that (see **Figure 1**):

Hypothesis 1: A perceived autonomy-supportive interpersonal style of the coach at the beginning of the first season (T1) will positively predict needs satisfaction in young footballers at the end of this season (T2).
Hypothesis 2: Needs satisfaction at the end of the first season (T2) will positively predict IM at the end of the first season (T2).
Hypothesis 3: IM at the end of the first season (T2) will positively predict subjective vitality at the end of the first season (T2).
Hypothesis 4: Needs satisfaction, IM, and subjective vitality at the end of the first season (T2) will positively predict these same variables at the beginning of the second season (T3).

## Materials and Methods

### Participants

The participants were 360 male footballers from Spain, ranging in age from 11 to 13 years (*M* = 12.60, *SD* = 0.52) and representing 40 teams from 26 different clubs registered in the Valencian Community Football Federation. Cluster sampling was used. The teams were randomly selected by geographic areas of the Valencian Community (Spain), using a list provided by the Football Federation. Ten of the 26 clubs contributed with two teams, two clubs contributed with three teams, and the rest of the clubs contributed with one team. Two hundred and eighty of the 360 players reported that the coach in the second season was not the same as in the first season.

Each team had only one coach responsible for training, and there was a different coach for each team.

### Procedure

A letter describing the purpose and characteristics of the study was sent to the managers and the coaches of the clubs. All the clubs contacted positively responded to the request. Subsequently, the coaches, the participants, and their parents were informed about the study aims and procedure, and they provided informed consent before data collection began. The study was anonymous and voluntary, and the usual ethics guidelines for research were guaranteed. The questionnaires were filled out at the different football clubs before one of their training sessions, and they took approximately 45 min to complete. A researcher explained the instructions for completing the questionnaires and resolved participants’ doubts. Neither the coaches nor the clubs’ managers were present during questionnaire administration. The first measure was carried out 2 months after the beginning of the first season, in order to allow the subjects to obtain information about the interpersonal styles of their coaches (Standage et al., 2003). The second measure was conducted at the end of that season. Finally, the third measure took place within the first 2 months of the next season. This research was conducted in accordance with international ethical guidelines, which are consistent with American Psychological Association guidelines. Ethical approval to conduct this study was obtained from the first author’s university ethics review committee.

### Instruments

#### Autonomy Support

The Spanish short version (Balaguer et al., 2009) of the Sport Climate Questionnaire (SCQ^1^) was used to assess players’ perceptions of the autonomy support provided by their coaches. This scale is composed of six items (e.g., “My coach offers me different alternatives and options”), each starting with the phrase: “On my soccer team…”and the responses are rated on a 7-point Likert scale ranging from 1 (*not at all true*) to 7 (*very true*). The scale has been used in the sport domain, and evidence of adequate reliability and validity has been obtained (e.g., Adie et al., 2008; Balaguer et al., 2009, 2012).

#### Needs Satisfaction

To evaluate satisfaction of the need for competence, the Spanish version (Balaguer et al., 2008) of the Perceived Competence subscale from the Intrinsic Motivation Inventory (IMI; McAuley et al., 1989) was used. To evaluate satisfaction of the need for autonomy, the Spanish version (Balaguer et al., 2008) of the Perceived Autonomy in Sport Scale (Reinboth and Duda, 2006) was used. The 10 items on the scale assess how players feel in general when they play football (e.g., “When I play soccer... I feel free to express my ideas and opinions”). The scale was composed of five items (e.g., “I think I am pretty good at playing football”). Finally, to evaluate satisfaction of the need for relatedness, the Spanish version (Balaguer et al., 2008) of the acceptance subscale from the Need for Relatedness Scale (Richer and Vallerand, 1998) was used. The subscale was composed of five items (e.g., “When I play football, I feel supported”). Responses were given on a 7-point scale ranging from 1 (*not at all true*) to 7 (*very true*). Previous studies in the sport domain have shown adequate reliability and validity (e.g., Reinboth and Duda, 2006; Balaguer et al., 2008, 2012).

#### Intrinsic Motivation

Intrinsic motivation of the athletes was evaluated with the Spanish version (Balaguer et al., 2007) of the three IM subscales of the Sport Motivation Scale (SMS; Pelletier et al., 1995). Specifically, athletes responded to the 12 items assessing IM to know (4 items, “For the pleasure of discovering new training techniques”), IM to accomplish (4 items, “For the satisfaction I experience while I am perfecting my abilities”), and IM to experience stimulation (4 items, “For the excitement I feel when I am really involved in the activity”). Each item starts with the phrase: “I play football…”, and the responses are rated on a 7-point Likert scale ranging from 1 (*not at all true*) to 7 (*very true*). Support for combining the three forms of IM into a single measure has previously been provided in the sport literature (e.g., Hollembeak and Amorose, 2005; Balaguer et al., 2007), and evidence for its reliability and validity has also been provided in the sport context (e.g., Balaguer et al., 2008; Álvarez et al., 2012).

#### Subjective Vitality

To assess the players’ feelings of energy, the Spanish version (Castillo et al., 2017) of the Subjective Vitality Scale (SVS; Ryan and Frederick, 1997) was used. The scale was composed of six items (e.g., “I feel alive and vital”) answered on a 7-point scale ranging from 1 (*not at all true*) to 7 (*very true*). The reliability and validity of this scale have been supported in previous studies conducted in the sport domain (e.g., Álvarez et al., 2012; Balaguer et al., 2012; González et al., 2015).

### Data Analysis

The validity of the scales was analyzed via confirmatory factor analysis (CFA) of the measurement models at the three different time points. Cronbach’s alpha was employed to assess the internal reliability of each scale. Descriptive statistics and bivariate correlations were also calculated. To examine the hypothesized model (**Figure 1**), due to the number of parameters in the model and the sample size, mean scores were used as indicators of the targeted variables, and a path model was tested. Furthermore, following the theoretical postulates of SDT (Ryan and Deci, 2000) and the procedures used in previous studies (e.g., Balaguer et al., 2012), we calculated the mean of the three basic psychological needs to assess the overall satisfaction of the three needs. To determine the fit of the models (measurement and path), several indices were considered that included chi-square, the comparative fit index (CFI), the incremental fit index (IFI), the non- normative fit index (NNFI), the root mean square error of approximation (RMSEA), and the standardized root mean square residual (SRMR). Values of CFI, IFI, and NNFI above 0.90 indicate an acceptable fit. For RMSEA and SRMR, values between 0.05 and 0.10 are considered acceptable. The analyses were performed using the statistical software SPSS and LISREL 8.80 (Jöreskog and Sörbom, 2006).

Because the coach in the second season is not the same one as in the first season, in order to test whether the effects on the variables at the end of the season (T2) remain at the beginning of the following season (T3), the perception of the coach’s autonomy support at the beginning of the second season (T3) was used as a control variable, including the effect of this variable on needs satisfaction at the beginning of the second season (T3). Moreover, additional paths were included in the model in order to cover the mediational effects of the variables in T3, that is, a path from needs satisfaction in T3 to IM in T3, a path from IM in T3 to subjective vitality in T3, a path from needs satisfaction in T2 to IM in T3, and a path from IM in T2 to subjective vitality in T3. All the variables in T1 were also included as control variables for their corresponding variables in T2, and so the following paths were added: a path from needs satisfaction in T1 to needs satisfaction in T2, a path from IM in T1 to IM in T2, and a path from subjective vitality in T1 to subjective vitality in T2.

## Results

### Measurement Models

The fit indexes of the CFA of the measurement models were acceptable in T1: χ^2^ (1319) = 4270.89, *p* < 0.001, CFI = 0.919, IFI = 0.919, NNFI = 0.916, RMSEA = 0.081 (90% CI = 0.079–0.084), SRMR = 0.074; in T2: χ^2^ (662) = 3715.15, *p* < 0.001, CFI = 0.902, IFI = 0.902, NNFI = 0.901, RMSEA = 0.081 (90% CI = 0.061–0.096), SRMR = 0.069; and in T3: χ^2^ (1268) = 4543.59, *p* < 0.001, CFI = 0.906, IFI = 0.906, NNFI = 0.901, RMSEA = 0.079 (90% CI = 0.079–0.082), SRMR = 0.072.

### Descriptive Statistics, Internal Consistency and Correlational Analyses

**Table 1** presents the means, standard deviations, and bivariate correlations between the variables used in the study. Cronbach internal reliability coefficients for all scales were satisfactory (alpha range = 0.78–0.93). In general, players reported moderate levels of needs satisfaction (competence, autonomy, and relatedness), IM, subjective vitality, and coach autonomy support. Based on the foundations of the SDT (Deci and Ryan, 1985), the results of the correlations were as expected, with positive and significant associations between all the variables.

**Table 1:** Means, standard deviations, Cronbach’s alphas, and bivariate correlations among the study variables.

Variable	1	2	3	4	5	6	7	8	9	10	11	12	13	14	15	16	17	18	19	20
1. CAS T1	1																			
2. CAS T3	0.17^∗^	1																		
3. SNC T1	0.16^∗^	0.02	1																	
4. SNC T2	0.11^∗^	0.03	0.55^∗^	1																
5. SNC T3	0.08	0.22^∗^	0.38^∗^	0.42^∗^	1															
6. SNA T1	0.50^∗^	0.09	0.30^∗^	0.21^∗^	0.14^∗^	1														
7. SNA T2	0.37^∗^	0.16^∗^	0.25^∗^	0.27^∗^	0.11^∗^	0.48^∗^	1													
8. SNA T3	0.21^∗^	0.46^∗^	0.10	0.09	0.20^∗^	0.27^∗^	0.41^∗^	1												
9. SNR T1	0.55^∗^	0.25^∗^	0.29^∗^	0.24^∗^	0.20^∗^	0.52^∗^	0.38^∗^	0.29^∗^	1											
10 SNR T2	0.43^∗^	0.20^∗^	0.16^∗^	0.38^∗^	0.12^∗^	0.31^∗^	0.53^∗^	0.23^∗^	0.53^∗^	1										
11. SNR T3	0.25^∗^	0.50^∗^	0.18^∗^	0.19^∗^	0.34^∗^	0.22^∗^	0.22^∗^	0.54^∗^	0.40^∗^	0.36^∗^	1									
12. PNS T1	0.56^∗^	0.15^∗^	0.58^∗^	0.38^∗^	0.27^∗^	0.89^∗^	0.51^∗^	0.30^∗^	0.77^∗^	0.43^∗^	0.33^∗^	1								
13. PNS T2	0.42^∗^	0.18^∗^	0.36^∗^	0.58^∗^	0.23^∗^	0.47^∗^	0.89^∗^	0.37^∗^	0.49^∗^	0.79^∗^	0.32^∗^	0.57^∗^	1							
14. PNS T3	0.24^∗^	0.53^∗^	0.24^∗^	0.25^∗^	0.55^∗^	0.29^∗^	0.37^∗^	0.88^∗^	0.38^∗^	0.31^∗^	0.80^∗^	0.39^∗^	0.41^∗^	1						
15. IM T1	0.38^∗^	0.12^∗^	0.23^∗^	0.17^∗^	0.18^∗^	0.39^∗^	0.28^∗^	0.18^∗^	0.34^∗^	0.21^∗^	0.17^∗^	0.43^∗^	0.30^∗^	0.23^∗^	1					
16. IM T2	0.33^∗^	0.09	0.20^∗^	0.32^∗^	0.19^∗^	0.31^∗^	0.44^∗^	0.25^∗^	0.27^∗^	0.43^∗^	0.19^∗^	0.35^∗^	0.52^∗^	0.28^∗^	0.52^∗^	1				
17. IM T3	0.23^∗^	0.28^∗^	0.15^∗^	0.20^∗^	0.28^∗^	0.16^∗^	0.23^∗^	0.48^∗^	0.19^∗^	0.18^∗^	0.41^∗^	0.21^∗^	0.26^∗^	0.53^∗^	0.41^∗^	0.47^∗^	1			
18. SV T1	0.27^∗^	0.13^∗^	0.29^∗^	0.20^∗^	0.25^∗^	0.35^∗^	0.24^∗^	0.22^∗^	0.31^∗^	0.12^∗^	0.20^∗^	0.41^∗^	0.25^∗^	0.28^∗^	0.53^∗^	0.33^∗^	0.33^∗^	1		
19. SV T2	0.21^∗^	0.12^∗^	0.13^∗^	0.24^∗^	0.15^∗^	0.23^∗^	0.37^∗^	0.32^∗^	0.29^∗^	0.32^∗^	0.24^∗^	0.28^∗^	0.42^∗^	0.34^∗^	0.32^∗^	0.48^∗^	0.36^∗^	0.46^∗^	1	
20. SV T3	0.16^∗^	0.23^∗^	0.14^∗^	0.17^∗^	0.19^∗^	0.09	0.17^∗^	0.34^∗^	0.17^∗^	0.18^∗^	0.40^∗^	0.16^∗^	0.22^∗^	0.41^∗^	0.21^∗^	0.25^∗^	0.50^∗^	0.37^∗^	0.50^∗^	1
Mean	5.38	5.24	5.63	5.64	5.69	5.04	5.00	5.17	5.73	5.60	5.82	5.36	5.31	5.47	5.71	5.51	5.43	5.49	5.52	5.52
*SD*	0.92	1.13	0.90	0.89	0.92	0.94	1.00	0.96	1.08	1.16	1.10	0.76	0.81	0.77	0.92	0.96	1.09	0.99	1.04	1.13
Alpha	0.89	0.93	0.74	0.77	0.79	0.79	0.84	0.86	0.87	0.90	0.92	0.86	0.88	0.89	0.89	0.90	0.93	0.78	0.81	0.85

*CAS, coach autonomy support; SNC, satisfaction need for competence; SNA, satisfaction need for autonomy; SNR, satisfaction need for relatedness; PNS, psychological needs satisfaction; IM, intrinsic motivation; SV, subjective vitality; T1, beginning of the first season; T2, end of the first season; T3, beginning of the second season. Range of variables = 1–7. ^∗^p < 0.05.*

### Path Analysis

The hypothesized model presented an adequate fit to the data: χ^2^ (31) = 98.427, *p* < 0.001, CFI = 0.968, IFI = 0.969, NNFI = 0.944, RMSEA = 0.078 (90% CI = 0.061–0.096), SRMR = 0.069. The results showed that a perceived autonomy-supportive interpersonal style of the coach in T1 positively predicted needs satisfaction in T2, which in turn positively predicted IM in T2; additionally, IM in T2 positively predicted subjective vitality in T2. Furthermore, after controlling for the perception of the coach’s autonomy support at the beginning of the second season (T3) because the coach changed from one season to another, the effects on the variables at the end of the season (T2) remained at the beginning of the following season (T3). Specifically, needs satisfaction in T2 predicted needs satisfaction in T3; IM in T2 predicted IM in T3; and subjective vitality in T2 predicted subjective vitality in T3 (see **Figure 1** and **Table 2**).

**Table 2:** Results for the path analysis.

Predictor	B	SE	*t*
**Outcome variable: Psychological needs satisfaction T2**		
Coach autonomy support T1	0.13	0.05	2.56^∗∗^
Psychological needs satisfaction T1	0.51	0.05	10.03^∗∗^
**Outcome variable: Intrinsic motivation T2**		
Psychological needs satisfaction T2	0.41	0.04	9.65^∗∗^
Intrinsic motivation T1	0.40	0.04	9.53^∗∗^
**Outcome variable: Subjective vitality T2**		
Intrinsic motivation T2	0.37	0.05	8.02^∗∗^
Subjective vitality T1	0.34	0.05	7.48^∗∗^
**Outcome variable: Psychological needs satisfaction T3**		
Coach autonomy support T3	0.47	0.04	11.02^∗∗^
Psychological needs satisfaction T2	0.32	0.04	7.64^∗∗^
**Outcome variable: Intrinsic motivation T3**		
Psychological needs satisfaction T2	0.15	0.05	2.85^∗∗^
Psychological needs satisfaction T3	0.46	0.04	10.16^∗∗^
Intrinsic motivation T2	0.42	0.05	8.62^∗∗^
**Outcome variable: Subjective vitality T3**		
Intrinsic motivation T2	0.15	0.05	2.95^∗∗^
Intrinsic motivation T3	0.42	0.05	8.99^∗∗^
Subjective vitality T2	0.42	0.05	8.88^∗∗^

*T1, beginning of the first season; T2, end of the first season; T3, beginning of the second season. ^∗∗^p < 0.01.*

Regarding the additional paths included in the model, needs satisfaction in T3 positively predicted IM in T3, which in turn positively predicted subjective vitality in T3. Finally, needs satisfaction in T2 positively predicted IM in T3, and IM in T2 positively predicted subjective vitality in T3.

## Discussion

The main objective of this study, grounded in self-determination theory (SDT, Ryan and Deci, 2000), was to test a longitudinal model in young male footballers. This model contemplated the four-stage sequence proposed by the HMIEM (Vallerand, 1997, 2001), focused on explaining variability in athletes’ subjective vitality. The model postulated that the perceived coach autonomy support at the beginning of the season would predict young football players’ subjective vitality at the end of the season, through needs satisfaction and at the end of the season, and that the effects on the variables at the end of the season would remain at the beginning of the next season. Results offered overall support for the proposed model, emphasizing the importance of athletes’ perceived coach-autonomy support at the beginning of the season because this kind of interaction has positive implications on young male athletes’ quality of leisure experiences and their well-being during the season.

With respect to the first hypothesis of the proposed model, results revealed that athletes’ perceptions of their coach’s autonomy support at the beginning of the season positively predicted needs satisfaction in young footballers at the end of the season. These findings are consonant with the proposed hypothesis and with previous research involving athletes (Bartholomew et al., 2011; Adie et al., 2012) and vocational dancers (Quested and Duda, 2011). This positive association is congruent with SDT, which holds that satisfaction of each of the three psychological needs is facilitated by autonomy support (Ryan and Deci, 2017). When athletes perceive that their coaches create this atmosphere from the beginning of the season, during the season they are more likely to feel like the originators of their own behavior, feel competent in their sport, and experience feelings of belonging.

Regarding the second hypothesis, which focused on the expected interplay between satisfaction of basic psychological needs and more intrinsic reasons for engagement, results showed that needs satisfaction at the end of the first season positively predicts IM at the same time in the season. According to SDT, satisfaction of psychological needs is the mechanism through which people move toward more self-determined motivation; that is, when young football players can freely choose to pursue the activity (autonomy), feel competent in their sport (competence), and feel respected and accepted, the reasons for participating in youth sport are more likely to be the inherent pleasure and satisfaction the activity provides. Previous studies have found relationships between needs satisfaction and IM in sport (e.g., Podlog et al., 2015) and in PE (e.g., Standage et al., 2006).

Also in agreement with SDT, and supporting the third hypothesis, results showed that when young athletes participate in football and enjoy the activity itself (IM), their eudaimonic well-being is promoted; that is, they feel full of energy and aliveness. Moreover, similar to previous studies that reported positive links between IM and subjective vitality in young female gymnasts (Gagné et al., 2003), young female tennis players (Balaguer et al., 2011), and young footballers (Álvarez et al., 2012), the present research suggests that intrinsic interest among boys involved in the sport of youth football is also tied to an indicator of positive eudaimonic well-being and optimal functioning.

Taken together, these findings agree with previous studies that tested the HMIEM (Vallerand, 1997, 2001) sequence in both cross-sectional (Amorose and Anderson-Butcher, 2007; Adie et al., 2008; Balaguer et al., 2008; López-Walle et al., 2012; Reynolds and McDonough, 2015; Haerens et al., 2017) and longitudinal study designs (Adie et al., 2012; Balaguer et al., 2012; González et al., 2017).

The findings of the current study also extend previous literature by demonstrating that the effect of players perceptions of the coaches autonomy support at the beginning of the first season, which has effects on the individual indicator variables of the quality of the experience and well-being at the end of the season, continues to have an effect at the start of the following season, regardless of the perceived coach autonomy support in the second season. Specifically, support for the fourth hypothesis was found, noting that needs satisfaction, IM, and subjective vitality at the end of the first season positively predicted these variables at the beginning of the second season. In other words, when the basic psychological needs have been satisfied during the season, the effect remains at the beginning of the following season because the young players still feel satisfied their needs of competence, autonomy, and relatedness, regardless of the degree of perceived autonomy support provided by their coaches at the start of the second season. The same thing has been observed for IM and subjective vitality; when players enjoy football for the inherent pleasure and satisfaction of the game itself, and they feel full of energy and aliveness during a season, the quality of the experience remains at the beginning of the next season.

Some implications may be derived from the findings of the current study. First, the explanatory model helps us to understand the associations between the perception of contextual variables (coach-autonomy support) and the internal motivational processes (needs satisfaction and IM) in young footballers over time. Moreover, the results inform us that the capacity to predict needs satisfaction, IM, and subjective vitality stems from the previous levels of these variables at the beginning of the season when athletes perceive that their autonomy is supported by the coach. These data reveal that the coach should adopt an autonomy supportive style from the beginning of the season in his/her interactions with the players because this atmosphere benefits the “full functioning” of the young athletes. When young boys play football surrounded by this motivational climate, they feel full of energy, both psychological and physical, to pursue their valued activities. It has been argued that developing this energy of self, considered as the experience of feeling alive, vigorous, and energetic, depends to a large degree on the motivational climate created by significant others, in our case the coach (Ryan and Frederick, 1997; Ryan and Deci, 2017). In this regard, it is essential to promote coaches’ autonomy-supportive interpersonal styles, not only due to the benefits announced in this study, but also because the literature has associated this interpersonal style with other positive consequences, such as being active (Fenton et al., 2017), the intention to be active and adherence to the sport (Álvarez et al., 2012; Quested et al., 2013; Cuevas et al., 2014), effort and satisfaction with the coach’s leadership (Álvarez et al., 2013), sport commitment (Pulido et al., 2018), or sportsmanlike attitudes (Sánchez-Oliva et al., 2016).

In terms of the study limitations, the first one is that all the information was obtained through self-reported measures. Although this procedure is widely supported by the literature and provides information of interest, it would be advisable to complement the perception of support for the player’s autonomy with measures that evaluate the real behavior of the coach in his/her interactions with the players, as in observational methodologies (e.g., Tessier et al., 2010; Smith et al., 2016; Fabra et al., 2018). Likewise, in order to further expand the knowledge about what motivational climate factors have a greater impact on the motivation and well-being of athletes, new longitudinal studies are necessary using a more recent theoretical approach (Duda, 2013; Duda et al., 2018) that integrates theoretical tenets and concepts stemming from achievement goal theory (e.g., Nicholls, 1989) and self-determination theory (Deci and Ryan, 1985). In addition, it should be emphasized that, although the data were obtained in a large sample, they are limited to the sport of football and young male grassroots football players (11–13 years), and so caution should be used in the extrapolation of the results. It would be worthwhile for future studies to confirm the relationships in both genders, in other age groups, and in different sports. Finally, it is important to emphasize that this study did not examine different dimensions of the social environment, and it is possible that other interpersonal behaviors (e.g., control) displayed by a new coach (at T3) would influence the association between autonomy support at T1 and needs satisfaction, quality motivation, and subjective vitality at T3. Future studies should analyze the consequences of the dimensions of disempowering climate (controlling style, ego involving climate) on the values of the individual variables (i.e., needs, IM, subjective vitality) over time.

At a practical level, the results of the current study emphasize the importance of using an autonomy-supportive interpersonal style where players can see their perspectives, values, and objectives respected. In this way, coaching behaviors based, for example, on acknowledging athlete’s feelings and perspectives, providing opportunities for initiative taking, minimizing external rewards, and offering meaningful information and rationales for requested tasks, will favor athletes’ full functioning through the mechanisms postulated in the HMIEM sequence (Vallerand, 1997, 2001). Given the findings obtained in this study, coaches interested in promoting quality in the leisure experiences and well-being of their athletes have the possibility of being trained to be more autonomy supportive (e.g., Duda et al., 2018), as previous studies in different contexts have found support for these types of interventions (e.g., Duda et al., 2014; Reeve and Cheon, 2016). This training fills a gap in coaches’ preparation because, as pointed out previously (e.g., Smith et al., 2007; Sousa et al., 2008), coaches’ training is mainly oriented toward technical and tactical aspects, leaving the creation of a positive, enjoyable and healthy atmosphere in the background.

## Author Contributions

All authors listed have made a substantial, direct and intellectual contribution to the work, and approved it for publication.

## Conflict of Interest Statement

The authors declare that the research was conducted in the absence of any commercial or financial relationships that could be construed as a potential conflict of interest.

## Footnotes

1 http://www.psych.rochester.edu/SDT/
